# How Can Transitional Housing Be Improved? Insights from Residents’ Experiences and Perceptions in New York City

**DOI:** 10.3390/ijerph21070829

**Published:** 2024-06-26

**Authors:** Zeynab Jouzi, Lauren San Diego, Neil A. Lewis, Tashara M. Leak

**Affiliations:** 1 Action Research Collaborative (ARC), Cornell University, Ithaca, NY 14853, USA; nlewisjr@cornell.edu (N.A.L.J.); tml226@cornell.edu (T.M.L.); 2 Division of Nutritional Sciences, Cornell University, Ithaca, NY 14853, USA; lds224@cornell.edu; 3 Department of Communication, Cornell University, Ithaca, NY 14853, USA

**Keywords:** homelessness, housing, social determinants of health, health equity, support services, urban health, transitional housing, lived experience

## Abstract

Homelessness, affecting over half a million Americans, significantly elevates the risks of mental and physical health issues, consequently diminishing life expectancy when compared with the general population. Homelessness is a critical public health issue, and efforts are needed to address lack of housing as a social determinant of health. Transitional housing (TH) programs emerge as vital interventions, offering a place to stay with various support services to facilitate the transition to permanent residency. Nearly half of the unhoused population in the country and over 90% in New York live in TH or shelters. Despite the high utilization rates of TH, engagement with support services and opportunities for improvement remain poorly understood. This study aimed to fill this gap by examining the factors influencing support service usage and opportunities for enhancement through semi-structured interviews with TH residents in New York City to capture their lived experiences and perspectives. Analysis of the interviews (*n* = 20) revealed five main factors affecting service engagement that aligned with constructs of the socioecological model: intrapersonal (self-efficacy, chronic health conditions, mental health), interpersonal (parenthood and well-being of children with special needs, individual staff interactions, and communication), institutional (bureaucratic challenges, administrative burden, and living facilities), community (social isolation and educational opportunity), and policy (challenge meeting basic needs and undocumented status). Recommendations for bridging service gaps primarily arose at the institutional and community levels, offering critical insights for administrators to tailor services more effectively to TH residents’ needs, thus contributing to the broader goal of advancing health equity among the unhoused.

## 1. Introduction

Over half a million Americans experience homelessness, a lack of fixed, regular, and adequate nighttime residence [1]. Homelessness is a public health crisis that undermines the physical and mental health of unhoused populations [2]. New York City (NYC) has the largest proportion of families with children experiencing homelessness in the US, with nearly half of NYC’s unhoused population consisting of families with children [1]. The negative effects of homelessness are not only concentrated among adults, but children are also harmed in numerous ways. Children who experience homelessness are more likely to fall sick, be diagnosed with emotional disturbances, and have worse academic experiences and outcomes [3].

In recent years, there has been an increasing acknowledgment of the importance for healthcare systems to better understand and measure social and economic factors to improve public health. These factors, known as social determinants of health (SDOH) encompass the environments where individuals are born, live, learn, work, play, worship, and age, and they have been associated with various health outcomes. Evidence suggests that SDOH may contribute more significantly to variations in health and health outcomes than traditional factors of healthcare systems. Among these determinants, housing is particularly fundamental, with homelessness posing significant social and medical vulnerabilities [4].

Although the literature has explored the link between housing and health outcomes, a deeper understanding of housing’s complexities is essential for mitigating health and social inequities [5]. To achieve health equity, where all people are given the support needed to achieve optimal health, tackling disparities in SDOH, such as housing, is crucial. Studies show that the disparities observed in those experiencing homelessness are not solely a result of individual choices but rather by SDOH [6,7]. Housing stands as a pivotal SDOH, with individuals experiencing homelessness representing some of the most socially and medically vulnerable groups in society [8,9].

Transitional housing (TH) programs are one response addressing housing as a SDOH. These programs offer people facing homelessness accommodation and supportive services for up to 24 months [1]. However, the dynamics influencing utilization of all TH services have not been fully elucidated. These programs are distinct from emergency shelters (ES), which are shorter-term and provide fewer intensive services, and safe havens (SH), which cater to individuals with higher needs, such as severe mental illness [1]. According to the US Department of Housing and Urban Development, TH programs facilitate the transition to permanent housing by offering a wide range of supportive services, which participants may be required to engage with as a condition of the program. These services can continue for up to 6 months after participants exit the program to assist with independent living.

Eligible supportive services include annual assessment of services, moving costs, case management, childcare, education services, employment assistance and job training, food, housing search and counseling services, legal services, life skills training, mental health services, outpatient health services, outreach services, substance abuse treatment services, transportation, and utility deposits [9]. The ‘Right to Shelter’ mandate in NYC, for instance, guarantees temporary shelter to the unhoused [10]. As a result, over 90% of NYC’s unhoused population is sheltered, the largest in the nation [1]. Therefore, NYC offers valuable insights for investigating the utilization of TH services for improving health equity.

This study aimed to examine the factors influencing support service usage and opportunities for enhancement through semi-structured interviews with adults with at least one accompanying child who resided at a NYC TH residence.

Specifically, we first asked to what extent the services provided in transitional housing engage with and benefit residents. Secondly, how do residents perceive service gaps? Finally, what solutions do residents suggest for bridging these identified gaps? During the interviews, it became evident that service utilization in TH was influenced by factors at multiple levels. Therefore, we adopted the social ecological model (SEM) as our theoretical framework for organizing and reporting our findings. The SEM, which has been utilized in other studies on homeless populations [5,7], provides a comprehensive framework for understanding how various levels of influence—intrapersonal, interpersonal, institutional, community, and policy—interact to affect health behaviors and outcomes.

Asking the TH residents about their suggestions for improving the services is particularly vital for marginalized groups, such as those experiencing homelessness, who are frequently excluded from decision-making processes [11]. Analyzing the perspectives of TH service users is essential for developing targeted interventions that can more effectively support those experiencing homelessness, ultimately contributing to a reduction in health disparities and progress towards health equity.

## 2. Materials and Methods

### 2.1. Study Site, Population, and Recruitment

This study was conducted at a TH facility located in Queens, NYC, which shelters 82 families with children. At the national level, nearly half (46%) of transitional housing beds are designated for families with children [1]. The facility, built in 1989, provides 40 single-family units equipped with kitchens and 21 double units (two families sharing kitchen facilities). Beyond offering housing, this TH site offers various support services (e.g., employment and education) and amenities (e.g., recreational areas and a children’s playground). Recognizing the dynamic nature of TH residency, we obtained a snapshot of the residents’ demographics from the TH database to ensure the representativeness of our sample. Inclusion criteria for this study were one adult (age 18 and above) per family living in the TH with at least one child and who could speak in English or Spanish. Residents whose primary language was not English or Spanish were excluded. Participant recruitment was conducted via English and Spanish flyers and several in-person visits by the lead researcher. During the screening process, participants indicated if they wanted to complete study activities in English or Spanish. Participants received $50 for completing study activities. This study was approved by Cornell University’s institutional review board.

### 2.2. Data Collection

This study included one-time, semi-structured, hour-long interviews and a short survey with participants that were conducted in English or Spanish. A native Spanish-speaking scientist conducted all of the Spanish interviews. Data collection took place between April 2023 and September 2023. The lead researcher developed the interview protocol, detailed in Table 1, which included 12 open-ended questions encompassing service utilization from the lens of SDOH, residents’ experiences in TH, methods of accessing resources, suggestions for improvement of the services, and inquiries soliciting ideas for life-enhancing initiatives. Interviews for the study were conducted via Zoom, providing participants with the flexibility to choose their own time and location. The lead author dedicated over a year as a volunteer visitor in the TH facility to build trust and gain a deeper understanding of the study site. During this period, she became convinced that all residents were comfortable with participating in virtual interviews. To mitigate potential technical obstacles, an iPad equipped with the necessary software was made available at the facility. Furthermore, a staff member was trained to assist participants with navigating the technology. Notably, none of the participants requested or utilized this assistance. To ensure accuracy, all transcripts were reviewed and edited by an undergraduate research assistant. For the Spanish transcripts, we utilized Google’s Cloud Text-to-Speech service [12] for transcription and subsequent translation into English. The Spanish-speaking interviewer verified the accuracy and reviewed the emerging themes.

The short survey included questions about demographics and public assistance usage. Housing insecurity questions from the Social Interventions Research and Evaluation Network (SIREN) of the University of San Francisco, California [13] were also included. Collection of demographic data provided a context by which the qualitative findings may be transferable to other settings [14]. Experienced TH staff reviewed the interview guide and short survey to ensure the clarity, relevance, and sensitivity to the participants’ experiences. After their interviews, participants were compensated with $50 cash as an incentive.

### 2.3. Data Analysis

We used an inductive thematic analysis approach for data analysis. Guided by Braun and Clarke [15], our thematic analysis encompassed six phases. In the initial phase, two coders (the first and second authors) familiarized themselves with the data by reading the transcriptions and jotting down their initial thoughts and insights. The second phase, open code generation, involved identifying and coding intriguing elements throughout the entire dataset.

Third, in theme exploration, open codes were compiled into emerging themes. Each coder individually identified the open codes and themes. The coders had weekly meetings to discuss instances of coding discrepancies and achieve a consensus in the themes. Fourth, both coders reviewed the themes and codes to ensure all the important data had been captured. Next, in the fifth step, the coders collaboratively defined the themes, discerning the ‘core’ or fundamental aspect of what each theme represented. This triangulation of codes and themes from dual coders contributed to the credibility of these qualitative results’ confirmability [14].

The sixth and final step encompassed selecting compelling quotations from the data, conducting a final analysis of these chosen segments, and relating the analysis back to the research question. The extended on-site time spent by the lead researcher, probes within the interviews, and the collection of fieldnotes further ensured that the analyses accurately represented the participants’ experiences and interpretations [14]. Microsoft Excel Online was used to organize the codes and themes. MAXQDA 22.8.0 was used to facilitate management of the data.

## 3. Results

### 3.1. Survey Findings

The study sample included 20 individuals (one person per family), including 15 interviews conducted in English and 5 in Spanish. The sample population represented about 24% of the TH’s total population (82 families). In total, we conducted 20 semi-structured interviews. The participants were predominantly women (85%), with an average age of 34 years. A majority of the residents (60%) identified as Black/African American, while 60% also confirmed Hispanic, Latino, or Spanish origin. Education levels varied, with half of the participants having completed high school. The demographic profile further indicated that 60% of the participants were born outside the United States. Regarding marital status, 65% were single. The majority of participants were actively seeking employment (55%) or employed part-time (30%). Usage of public assistance programs (federal programs administered by states) was high, with 75% using the Supplemental Nutrition Assistance Program (SNAP); 40% using the Special Supplemental Program for Women, Infants, and Children (WIC); 20% using Temporary Assistance for Needy Families (TANF); 60% using Medicaid; 30% using Medicare; and 10% using the Children’s Health Insurance Program. Engagement with TH services was notably high for case managers and social workers (90%), housing specialists (85%), employment specialists (75%), and education specialists (40%). Our findings revealed diverse living situations prior to TH. However, a majority of the respondents had had stable living conditions in the past year and felt confident about maintaining their current housing for the next 2 months. The participants’ demographics, service utilization, and housing profiles are detailed in Table 2. These questions provided a clearer understanding of the people’s housing situations, enabling us to better interpret the results by comprehending the housing stability and living conditions of the study population prior to and during their stay in TH.

### 3.2. Interview Findings

Using the social ecological model (SEM) as a framework to organize the findings, our analysis of the interviews fit into five overarching themes (intrapersonal, interpersonal, institutional, community, and policy) which encompassed 13 sub-themes. This organization shed light on the intricate dynamics between personal experiences and broader societal influences on TH residents’ utilization of services. The subsequent sections delve into each theme and its corresponding sub-themes, providing a focused discussion on the various factors influencing service utilization. Furthermore, the suggestions offered by interviewees to enhance service utilization were discussed.

#### 3.2.1. Theme: Intrapersonal

Several sub-themes representing intrapersonal factors, or the residents’ particular characteristics, influenced the ways the residents interacted with TH services: self-efficacy, chronic health conditions, and mental health.

##### 3.2.1.1. Self-Efficacy

Residents’ perceived sense of autonomy to navigate the challenges encountered in TH services varied and factored in whether they obtained the support needed. Those demonstrating greater confidence in navigating the challenges encountered in TH services tended to have longer work or education histories. However, many residents cited their children as motivation for taking action to overcome the challenges encountered when navigating TH. For example, Interviewee #13, a pregnant resident who aimed to leave TH before giving birth, shared her efforts in securing permanent housing:

“I did do the fair hearings. I did write the letter to the mayor. I just never sent the email because I found another option online.”

##### 3.2.1.2. Chronic Health Conditions

Both the residents’ and their children’s chronic health conditions augmented challenges in living within the TH and their ability to take advantage of the services offered. For example, Interviewee #5 (34 years old, undocumented, mother of one) shared the health challenges faced by her son, who was diagnosed with a blood condition that was sensitive to extreme temperatures. She emphasized the importance of maintaining a suitable environment, especially during winter when heating was inadequate. Another resident, Interviewee #6, a mother of one who was taking medication for depression and anxiety described:

“My baby has breathing problems just as I do. I have asthma. I have breathing problems. Sometimes I have to open my door, and they would come and lock my doors.”

Interviewee #14, a 47-year-old undocumented single mother of a teenager, shared her recent diagnosis with diabetes and the resulting health complications. Fearful of the implications of her illness on her son’s well-being, she avoids insulin and rejected knee surgery, worried about job prospects and her ability to work. Despite her deteriorating health, including neuropathy in her feet that severely limits her mobility and causes intense pain, she maintains a facade of wellness at work. She is grateful for the basic healthcare and medication provided by her primary care doctor, which helps manage her pain, though her condition continues to challenge her daily life. She conceals the severity of her situation from her son, wanting to shield him from worry, and struggles with the high cost of necessary treatments, including one that was canceled due to its prohibitive expense.

##### 3.2.1.3. Mental Health

Finally, the nature of living in TH strained both residents’ and their children’s mental health. Interviewee #12, a 21-year-old mother, explained:

“Mental health. That’s a big thing here. They can have all the resources, but mentally, sometimes people need help in that area. That’s really important because some people know what to do and have a plan. But they just mentally are stuck, and they can’t do it. So, I would use resources. Because it’s really underestimated how much it helps. Honestly.”

Many residents were overwhelmed by the mix of challenges in managing parenthood, employment, health, immigration, and language barriers within the context of TH. For some, this inhibited their self-efficacy, resulting in under-utilization of the services offered.

#### 3.2.2. Theme: Interpersonal

Within the interpersonal sphere, residents’ immediate relationships with others affected their self-identity, priorities, and actions taken within TH. Sub-themes that emerged included parenthood and well-being of children with special needs, individual staff interactions, and communication.

##### 3.2.2.1. Parenthood and Well-Being of Children with Special Needs

Parenting within TH brought unique challenges, particularly for those who were single or first-time parents. Many residents felt that there were numerous improvements needed to better support the challenges of single and first-time parents. Interviewee #6 described her post-partum experience:

“I had to get up, my [Cesarean section] cut open back because the shelter had nobody to even give me a glass of water or to help me for the baby, I had no help.”

The need for support was especially poignant for those who had children with special needs. “So, my son has autism, and it’s my first time going through this process; and so a lot of it is new to me, and I’m not fully aware of what, you know, what he has access to, what’s available to him, what he is eligible for, and what the process is or overall steps is to ensure I get everything I need and everything available to him”, described Interviewee #3, a mother and a survivor of domestic violence.

Meeting these children’s needs and ensuring their well-being were frequently the primary priority, but parents reported inadequate support once their child was identified as having special needs.

##### 3.2.2.2. Staff Interactions

Staff heavily influenced the residents’ experiences in TH and their motivation to utilize services. Positive interactions that built positive relationships with individual staff members were cited as sources of inspiration and support. As a result, the residents’ self-efficacy improved, which translated into improved resiliency and facilitated effective utilization of TH services. Interviewee #12 shared:

“[My social worker] made me believe that it was possible for me; and, yeah, now I have my apartment and I’m almost out of here. I got my license… So, yeah, it was definitely her. I’d say… Every single time I go there, she’s just like, ‘I’m proud of you, I can’t believe you’ve come this far…’ Those little words… Even when she first said it to me, I wasn’t believing it, but then she planted a seed in my head.”

Conversely, interactions with individual staff members that fostered a sense of disrespect, callousness, and apathy inhibited the residents’ willingness to take part in the available services. Interviewee #5 described:

“She really spoke down on me, and it was so bad I was crying the whole day… I’m no longer going to, you know, participate in any services in this place because of that particular experience with that woman.”

Additionally, staff turnover inhibited the residents’ ability to form positive relationships with the staff.

##### 3.2.2.3. Communication

Communication challenges with staff also hindered the residents’ ability to utilize services, contributing to poor relationships with individual staff members and negatively impacting the residents’ mental health. This was especially true for Spanish-speaking residents, as they were constrained in the number of staff with whom they could interact and develop working relationships. Interviewee #8 described her frustration:

“I want to talk to [my social worker], but she talks and talks and puts on a translator, and nothing happens.”

In addition to having multiple modes of communication via flyers and intercom announcements, direct verbal communication about the services to the residents by the staff were valued not just by Spanish-speaking residents but by English-speaking ones as well. Regardless of language barriers, many residents found limitations in the bulletin boards and intercom system used as a central communication method, citing numerous ways to update this “outdated” system.

#### 3.2.3. Theme: Institutional

Factors within the TH site that also influenced the residents’ experiences and ability to engage with the services represented the institutional themes. The residents primarily described issues with bureaucratic challenges, the administrative burden, and living facilities.

##### 3.2.3.1. Bureaucratic Challenges

Many residents cited challenges in accessing the available services due to the bureaucratic obstacles they encountered, whether this was related to system overload or getting sent down a chain of referrals to meet a simple need. Interviewee #20 detailed her experience:

“My biggest problem is that I haven’t been able to get my daughter a school. [The social worker] told me, ‘Go to the school specialist’. But the school specialist told me that she had to call [the school offices]. They didn’t give me a solution; they just told me, ‘Call and ask’. I called, but they didn’t give me a solution. They tell me that I have to go here, then they tell me that they are going to help me, that I have to go to school in person, but when I go to school in-person they tell me, ‘No, you need a voucher’. But I can’t apply for a voucher because I don’t have a job. There are many things for me to get the girl’s school.”

Though some residents took initiatives to overcome these challenges, others remained stuck, unable to access the services.

##### 3.2.3.2. Administrative Burden

Furthermore, many residents commented on a sense that the TH staff fostered an attitude that looked down upon the residents. This pervasive attitude was characterized by a lack of respect, empathy, and competence, perceived not as isolated instances from individual staff but as a systemic issue. Interviewee #19 described:

“They talk down to you… They stereotype you because they think ‘Oh, you’re in a shelter. Everybody that comes in a shelter is a nobody’, and that is far from the case, you know. And sometimes things can happen to just about anybody. I think they just lack that respect for the residents.”

Multiple Spanish-speaking residents expressed a sense of discrimination by the staff. This not only discouraged the residents from utilizing services but also, in one case, placed an undue financial burden that inhibited her ability to obtain the other services needed. The Spanish-speaking resident, who, due to linguistic barriers and unfamiliarity with the local health system, felt unwell and was subjected to an unnecessary ambulance call by the staff without her consent. She described that the situation was not an emergency and that a taxi would have been a more appropriate, cost-effective option. The high cost of the ambulance service forced her to divert funds she had painstakingly saved for her asylum case attorney to cover the bill.

##### 3.2.3.3. Living Facilities

Though residents expressed gratitude for their place in TH, the physical aspects of the living facilities contributed to a sense of discomfort and negatively affected mental health. A lack of proper seating or uncomfortable sleeping arrangements chipped away at their well-being and contributed to an overall sense of despair. Interviewee #19, a single mother, elaborated:

“[The environment] is going to end up eating you out because you already… want [to] get going, and you want to sleep on a couch—a little bit of a comfortable bed, not luxury. Or you want to come home and not sit on the floor, but somewhere where you can just have a seat there and do something.”

Interviewee #2, a single father of two, shared a similar sentiment:

“After a while, because it kinda feels like you’re in jail. I’m in a one room cell with two kids.”

Perceptions of limited accessibility to certain amenities and challenges inherent to old buildings wore on already stressed and overwhelmed residents attempting to establish stability in their situations. Specifically, issues with furnishings, heat, and roach infestations were detrimental to the residents’ sense of safety and psychological well-being.

#### 3.2.4. Theme: Community

The theme of community encompassed the resources surrounding the TH and provided the context for health, social networks, and norms. Two sub-themes of social isolation and educational opportunities emerged.

##### 3.2.4.1. Social Isolation

Participants reported social isolation and a lack of support in their own social networks and reliance on the TH staff to fill these gaps. Interviewee #6 explained:

“Not everybody has family here, or has somebody they can turn to, and being in the shelter is very stressful. When you’re not getting the support you need, it’s even more stressful.”

Social isolation compounded the challenges residents encountered in navigating the TH system. Residents distrusted one another and did not have the bandwidth to take on the emotional burden of other relationships. There were a few residents who reported using their social network outside of the TH system for information and resources. However, many undocumented residents reported that their lack of a social network limited their ability for outside support beyond the TH.

##### 3.2.4.2. Educational Opportunities

Several residents expressed the desire to start or complete their education to improve their life circumstances and reduce the likelihood of returning to their current situation. However, they found a lack of community resources to achieve this goal. For example, Interviewee #2, a 42-year-old single father of two, articulated how expanding education reduces the likelihood of individuals reverting to old unhelpful patterns by broadening their knowledge base. Balancing the challenges of remote and in-person schooling with childcare, he views education for adults as crucial, ranking it as his second priority after his children’s needs. He critiqued reliance on temporary aid, such as vouchers, as insufficient for long-term improvement, advocating instead for educational opportunities that lead to sustainable employment and careers, not just hourly jobs. Interviewee #11, another father, mirrored the sentiment:

“All the time I spent here not doing anything, I’d rather have been educating myself and learning something.”

#### 3.2.5. Theme: Policy

Larger local, state, and federal policies determined the resources residents could practically access. These policies resulted in the residents having challenges meeting their needs. Residents who were undocumented faced additional challenges.

##### 3.2.5.1. Challenges Meeting Basic Needs

Some residents disclosed that they had difficulties feeding themselves and their families. Interviewee #16, a Spanish-speaking single mother of two, described the limits to the resources she could access:

“They give us food but… two servings… Why two small plates… What if there are three in the family? And if they are immigrants, if they don’t have a job, what are they going to eat at night? Then they will have to save half of that portion to eat at night.”

As a single mother seeking work, Interviewee #17 shared similar thoughts on limits:

“Most times, I can’t afford to get milk for my baby, cause he’s a milk lover. I got WIC [Special Supplemental Nutrition Program for Women, Infants, and Children], yeah, but WIC doesn’t give me enough.”

Several participants mentioned that they applied to programs such as SNAP and WIC. However, they were not yet receiving the benefits, leaving them food-insecure.

##### 3.2.5.2. Undocumented Status

Various policies placed additional hurdles for residents who disclosed that they were undocumented residents and augmented the challenges of living in TH. For example, without a green card, residents had no clear path to fulfill their aspiration for more education. Without a social security card or work permit, employment may be barred. Without employment, assistance for permanent housing such as vouchers or approval for public housing were rarely obtained. The lack of income from employment and permanent housing assistance made it exceedingly difficult for the residents to transition out of the TH. When seeking legal assistance, those who were undocumented were frequently left waiting at the mercy of the administrative processes by which they could obtain resources. Interviewee #14 voiced her frustrations:

“All of them require ‘Are you legal to work in America?’ First thing, it’s no, because I cannot say yes. That means I’ll be lying, and then they require the social [security card] which I don’t have. So, it put me on the back burner again… I know it’s maybe bad to them that I don’t have my documents. But I came to make a better life, and I want to do the right thing.”

#### 3.2.6. Recommendations for Bridging Service Gaps

Many of the residents’ identified service gaps and suggestions for improvement fell within the institutional and community levels of the SEM. There were several opportunities identified to improve the institutional factors of TH. Residents wanted the TH to modernize the communication system to effectively reach residents. Interviewee #17 explained the value of text messages:

“Everybody’s got a cell phone. And little notes, ‘cause it’s not everybody like me who stops and looks at the notice board. I read flyers, but once you get a text, you’re gonna look.”

Utilizing various methods of communication and providing opportunities to verbally share information were cited by multiple residents as ways to improve access to the current TH resources. Additional recreational activities for both adults and children were listed as one way to positively impact mental health and well-being, alleviating the sense of confinement experienced in shelters.

Improving the capacity to provide specialized support for first-time parents and parents of children with special needs was another type of support residents sought. Interviewee #3 appreciated the current staff’s efforts but sought more direction:

“You know Ms. B [one of the staff] has been a great help with trying to find that information [resources for parents of children with autism], but she as well isn’t specialized in knowing what to do as well. I know she’s trying to find people who can possibly come in, so we are working on subsidizing that. But as of right now, we don’t really have one.”

First-time single parents who lacked a personal support network wanted guidance from TH services. Interviewee #18, a single mother, suggested:

“Showing them what to do as a first-time mom. Like CPR, how to change their diapers, how many times like you should feed them a day.”

Interviewee #10, a single mother seeking asylum, prioritized single parents of children with disabilities:

“For me the priority would be mothers or fathers alone with children… or disabled children in this case that their mothers do not have the help. I would do it for them. Well, I would try to focus more on it. Try to get them the help they need.”

She further emphasized the importance of emotional support:

“Try to support them more emotionally because sometimes it’s not all economic. Sometimes I think that not everything is money because in life, not everything goes with money. Sometimes you also need a person who motivates you emotionally.”

Interactions with TH staff members could be improved through enhancing the competencies of understanding and respect for residents’ diverse backgrounds. Work in the context of social services goes beyond mere transactional interactions; it encompasses building meaningful, empathetic connections with the residents. Interviewee #8, a Spanish-speaking single mother, noted:

“Small improvements could be made, not even by investing a lot of money, but rather by dealing with the intention of helping… something beautiful, yes as a little bit of human warmth.”

Outside the TH institution, many residents expressed their belief that community partnerships were underleveraged. Regarding education and employment opportunities, several residents identified local resources to connect with TH services. Interviewee #8, a single mother facing barriers with undocumented status, explained:

“I studied about four semesters of social work in [my own country], and I didn’t finish, well due to my economic issues, but I know that they have resources, at least they can contact universities.”

Interviewee #13, an expecting mother, noted:

“I would look into the non-profits. Basically, that the shelter could partner with to make it a little bit more convenient, as far as the budget goes, to still get everything done.”

Leveraging connections with universities and collaboration with non-profits were thought to provide more resources such as short courses, certification programs, and psychological services. Additionally, residents saw that TH services could optimize employment services by proactively matching the residents’ skills and backgrounds with suitable job opportunities.

The residents expressed the need for specialized mental healthcare beyond what TH currently offered to support individuals during challenging transitions of being homeless and the associated struggles of finding stable housing, as they found the current systems inadequate. Interviewee #15, a single mother of two working full-time, noted:

“Honestly, the [staff] here aren’t equipped for that. They can, you know, tell you, talk to a case manager or take it up; but sometimes people need someone, you know, equipped with tools or licensed professionals, because this is a tough transition”

Interviewee #13 suggested exploring options for free counseling that were easily accessible to TH residents:

“How lawyers do pro bono cases, and they do a certain amount, like, you know, you’ll find like a specialist that can come in once a week.”

Residents with challenges related to their undocumented status expressed the need for access to legal assistance to improve their situation. Interviewee #14, a single mother struggling with multiple health challenges, emphasized this need:

“How can I get legal advice? Because I don’t wanna be in a shelter forever. I wanna make something out of my life… It’s a tough time. So, as I said, the best thing I can ask for is legal advice. How can I get a permit [to work]?”

These residents found that this gap in legal assistance was a major barrier to effectively utilizing the existing TH services.

Strengthening TH partnerships with organizations frequently interacted with by the residents is thought to facilitate more efficient interactions between the residents and these external agencies. One TH resident, Interviewee #19, who had over a decade of working with social services, explained:

“Your clients have to deal with HRA [The Human Resources Administration or Department of Social Services]. You know they probably have to deal with DHS [Department of Human Services], PATH [Prevention Assistance and Temporary Housing], whatever it is. You know, social security, stuff like that. It does not hurt for you to have a contact person at one of these facilities to say, ‘I’m having this issue. How do I resolve it? My client is going through this, you know. How do I resolve it?’ That is very limited. I see very limited communication between, or knowledge about the different entities and stuff like that.”

Interviewee #13 described attending an off-site program for support beyond what was offered in TH:

“Well, there is a program… It’s based off of like substance, or like a history of substance abuse. They’re basically focused on getting your transition and keeping you clean and making sure that you don’t [relapse] and stuff like that. That’s currently a program that I’m doing at another shelter.”

Finally, residents emphasized the need to ensure the consistent availability of food and essential items as a primary priority. While programs such as SNAP and WIC assist low-income families, undocumented immigrants are ineligible for most state and federal benefits. Food donations are also crucial for TH residents but are often untimely and insufficient, leading to potential food insecurity. Interviewee #4, a mother of two, described:

“Food will be the top… like more free items can be distributed… have at least a certain amount set for a food budget for families that don’t have food stamps or the case gets messed up.”

Meeting these fundamental needs was considered essential to improving the residents’ experiences in TH.

## 4. Discussion

This research explored the multifaceted nature of service utilization within TH, guided by the lived experiences of its residents. Our findings revealed several key insights, underscoring the necessity of adopting a multilevel approach to comprehensively understand service utilization in TH. The identified themes span from bureaucratic hurdles to personal mental health issues and the struggles with social isolation, shedding light on the varied and intricate lives of TH residents. Using a qualitative analysis in line with Braun and Clarke’s [15] approach, this study aimed to delve into the spectrum of experiences and challenges faced, rather than seeking to provide a definitive answer.

Our findings at the intrapersonal level underscore the importance of self-efficacy and mental health, aligning with the literature that identifies these as key factors in homelessness interventions [16]. The complex nature of homelessness, arising from socioeconomic factors such as poverty and unemployment, calls for systemic interventions for prevention. Simultaneously, addressing the psychological traits and resilience of individuals is critical to help individuals leave this situation. Enhanced personal skills and psychological support can promote autonomy and self-efficacy, which are often impaired in unhoused individuals, resonating with the concept of building resilience and self-sufficiency to help individuals adapt to and overcome adversity [16].

On an interpersonal level, our findings about the importance of interactions with staff align with studies highlighting the role of relationships in utilization of services by the unhoused population [17]. Viewing the relationship as the catalyst underscores the need for positive interactions between staff and residents. Echoing the findings of Rofle et al. [8], which acknowledged the profound impact of housing as a SDOH, highlighting the importance of quality relationships with housing providers, the residents’ perceptions of housing quality, and the presence of social support in influencing health and well-being. It is crucial to also consider the challenges faced by the staff themselves, such as high rates of burnout and post-traumatic stress disorder stemming from the complexities of working with this population [18]. Enhancing training and support for the staff is therefore a promising strategy to improve the delivery and utilization of services, enhancing the well-being of both the staff and the residents.

Existing literature that delves into the nuanced dynamics of homelessness and societal attitudes supports our findings at the institutional level regarding the administrative burden and poor living facilities. Homelessness is frequently seen as a condition within an individual’s control, resulting in a perceived legitimized form of discrimination. This stigmatization leads to the dehumanization of those experiencing homelessness. The negative treatment and living conditions they endure can be seen as a reflection of broader societal attitudes that devalue and marginalize them [19]. Moreover, our findings align with those of Lee et al. [20], who studied the homeless population’s experiences of services in Albany, NY, and revealed that individuals experiencing homelessness encountered discriminatory attitudes and demeaning interactions with the staff. Similarly, the results of Lee et al. [20] highlighted bureaucratic challenges, including poor service coordination, staff shortages, miscommunication, and rigid and often punitive policies, alongside infrastructural deficiencies such as outdated technology, which cumulatively led to prolonged waiting times and significant mental stress for service users.

In the community sphere, our findings on social isolation resonate with Malden et al. [21], who highlighted that social isolation and stigmatization significantly afflict the homeless population, frequently resulting in depression and self-harm. Their research evaluating a community-based intervention aimed at improving the health and well-being of homeless individuals through physical activity and peer support showed significant positive outcomes, including enhanced self-esteem, increased social interaction, and better health behaviors among the participants. These findings underscore the importance of addressing social isolation among the homeless to improve their overall well-being, mirroring our finding on the barriers to service utilization caused by social isolation.

Finally, at the policy level, our findings underscore the critical challenges undocumented individuals face, notably how the absence of legal documentation exacerbates challenges accessing employment and basic necessities such as food. This finding is aligned with Chang’s [22] study that highlighted food and housing insecurity among this population. Moreover, undocumented immigrants often avoid seeking necessary healthcare out of fear of arrest or deportation, resulting in underutilization of the available services [22].

Residents also offered suggestions at different levels to improve the services, ranging from enhancing communication to partnering with universities. Aligning with the residents’ feedback on communication and the need for modernization, Asgari et al. [23] found NYC shelter residents highly valued text messages for healthcare. Succinct, positive texts were preferred, indicating that tailored communication strategies could significantly aid this population. Our findings from the residents’ suggestions echo the call for increased collaboration among universities, non-governmental organizations, and other entities to bolster support and services for the homeless, resonating with insights from Mosley [24]. The US homeless service system’s fragmentation and resource scarcity have entrenched ineffective policies and interventions. This scenario, described as a “wicked problem,” underscores the urgency of cross-sector collaboration, which requires leadership committed to a shared vision and an equitable distribution of power, as highlighted by Mosley [24], to navigate the complexities of homelessness effectively.

The findings should be interpreted with consideration of certain limitations, including the study’s focus on a single site in Queens, NYC. The decision to concentrate on only one center was driven by the belief that deep, immersive, on-site engagement with the participants would yield more nuanced insights into the lived experiences of TH residents, prioritizing depth of understanding over the breadth of cross-site comparisons. Such depth is crucial for uncovering the complex dynamics of service utilization, resident–staff interactions, and the impact of institutional policies on individuals’ lives. Future research could include longitudinal studies across other sites for a broader understanding of residents’ experiences in TH.

## 5. Conclusions

Though homelessness is frequently depicted as a crisis in the media, the lived experiences of people experiencing homelessness are often overlooked. This research centered on the voices of those directly affected by homelessness. The findings of this study are important not only for enriching our understanding of TH residents’ experiences but also for their potential to inform targeted interventions. By identifying the specific needs and barriers faced by residents, this research sets the stage for more refined, effective TH service strategies, ultimately aiming to improve health equity and the residents’ well-being. From a research perspective, this study adds to the field of health equity for unhoused populations by viewing how TH services address housing as a SDOH. This approach highlights the need for holistic, integrated strategies in addressing health disparities among the homeless. Future research could include longitudinal studies across other sites for a broader understanding of residents’ experiences in TH.

## Figures and Tables

**Table 1:** Questions used to guide the semi-structured interviews.

To start, can you tell me a little about you and your family experience of living here? How do you learn about the resources available for you (flyers, words of month, emails, texts)? How can [this shelter] improve their methods of communication? Can you suggest a better way? Please tell me about your experience of working with the case manager here. What kind of health services do you have access to? What kind of employment services do you have access to? What kind of housing services do you have access to? What kind of education services do you have access to? Imagine that you are a manager and want to improve the life of people here. You have a limited budget, so you cannot do everything. You need to prioritize. What would you make your top priority? What is the best service you have received during your time here? What service/resource was the least helpful? Is there anything else that you would like to talk about?

**Table 2:** Demographic characteristics, service utilization, and housing situation of the study participants (*n* = 20).

Characteristic	Detail	% Sample
Age	Mean, 34 years; range: 21–55 years	
Gender	Women	85%
	Men	10%
	Gender not disclosed	5%
Educational levels	High school	50%
	Some college	35%
	Middle school	5%
	Associate degree	5%
	Bachelor’s degree	5%
Race	Black/African American	60%
	White	5%
	American Indian/Alaskan Native	5%
	Other (mixed race, unspecified)	30%
Hispanic, Latino, or Spanish origin	Yes	60%
	No	40%
Birthplace	Outside the United States	60%
	In the United States	40%
Language of interview	English	75%
	Spanish	25%
Marital status	Married	15%
	Widowed	-
	Separated	15%
	Divorced	5%
	Single	65%
Duration of residence in the study site	Range: 6 weeks to 2 years	-
Employment status	Actively seeking employment	55%
	Employed part-time	30%
	Employed full-time	10%
	Not seeking employment	5%
Public assistance program use	Supplemental Nutrition Assistance Program (SNAP)	75%
	Women, Infants, and Children (WIC) program	40%
	Temporary Assistance for Needy Families (TANF)	20%
	Medicaid	60%
	Medicare	30%
	Children’s Health Insurance Program	10%
Engagement with transitional housing services	Case managers and social workers	90%
	Housing specialists	85%
	Employment specialists	75%
	Education specialists *	40%
**Living situation prior to transitional housing**		
Temporarily in a shelter or homeless situation		30%
Temporarily staying with a relative or friend		25%
Living alone in own home (house, apartment, condo, trailer, etc.)		20%
Living in a household with other people		20%
Other living situations		5%
**Stable living conditions in the past 12 months**		
Stable (not staying outside, in a car, tent, or shelter, or couch-surfing)		70%
Experienced housing instability		30%
**Confidence in maintaining stable housing**		
Confident in maintaining stable housing for the next 2 months		60%
Concerned about losing stable housing in the next 2 months		40%

* Focusing on school-age education and thus not used by families with younger children.

## Data Availability

The original contributions presented in the study are included in the article, further inquiries can be directed to the corresponding author (Z.J).
